# Inflammation-targeted cannabidiol-loaded nanomicelles for enhanced oral mucositis treatment

**DOI:** 10.1080/10717544.2022.2027572

**Published:** 2022-04-25

**Authors:** Yingke Liu, Xingying Qi, Yashi Wang, Man Li, Quan Yuan, Zhihe Zhao

**Affiliations:** a State Key Laboratory of Oral Diseases, National Clinical Research Center for Oral Diseases, West China Hospital of Stomatology, Sichuan University, Chengdu, China; b Key Laboratory of Drug-Targeting and Drug Delivery System of the Education Ministry and Sichuan Province, Sichuan Engineering Laboratory for Plant-Sourced Drug and Sichuan Research Center for Drug Precision Industrial Technology, Sichuan University, Chengdu, China

**Keywords:** Oral mucositis, inflammation, cannabidiol, P-selectin, nanomedicine

## Abstract

One of the most common complications of cancer chemotherapy is oral mucositis (OM), a serious kind of oral ulceration, but its effective treatment remains a serious challenge. In this study, we used deoxycholic acid and fucoidan to prepare inflammation-targeting nanomicelles (FD), because fucoidan can target inflammation due to its high binding affinity for P-selectin. The hydrophobic anti-inflammatory drug cannabidiol (CBD) was then loaded into the hydrophobic core of FD. The resulting CBD-loaded FD micelles (CBD/FD) had uniform particle size and morphology, as well as favorable serum stability. Moreover, administration of the FD micelles *via* intravenous injection or *in situ* dripping in an OM mouse model enhanced the accumulation and retention of CBD. CBD/FD also showed a better anti-inflammatory effect compared to free CBD after local or systemic administration *in vivo*, while they accelerated OM healing and inhibited Ly6G inflammatory cell infiltration and NF-κB nuclear transcription. Our results show that CBD/FD nanomicelles are a promising agent for OM treatment.

## 1. Introduction

Oral mucositis (OM) is serious mucosa ulceration that commonly develops as a side effect of radiotherapy and chemotherapy (Luo et al., 2019). OM occurs in 20–40% of patients receiving normal chemotherapy and 80% of patients receiving high-dose radiotherapy as a pretreatment for bone marrow transplantation. Moreover, almost all patients receiving radiotherapy for neck and head cancer develop OM (Blakaj et al., 2019; Elad et al., 2020). OM can cause severe pain and affect swallowing and mastication, which not only reduces life quality but also negatively affects prognosis caused by reducing the dose, causing a heavy economic burden (Elad & Yarom, 2019; Pulito et al., 2020). Palifermin is currently the only drug authorized by the US Food and Drug Administration and the European Medicines Agency to treat OM (Spielberger et al., 2004; Elad et al., 2020). Guidelines also recommend treatments such as benzedrine mouthwash, cold therapy, laser therapy, and morphine pain-relieving (Riley et al., 2016; Sio et al., 2019; Elad et al., 2020). However, many of these recommendations are not implemented in the clinic because of insufficient or conflicting evidence about efficacy.

Cannabidiol (CBD) is extracted from *Cannabis sativa* plants and exhibits no psychoactivity, in contrast to Δ9-tetrahydrocannabinol from the same plant. CBD has shown promising pharmacological effects in several areas including neurological disorders, immune and cardiovascular diseases, multiple sclerosis, and cancer (Pisanti et al., 2017; Pacher et al., 2020). Promising therapeutic effects have also been observed in inflammatory areas including arthritis, inflammatory bowel and lung disease, and chemically-induced colitis (Burstein, 2015; Atalay et al., 2019; Dos-Santos-Pereira et al., 2020). Many of the effects are due to the ability of CBD to suppress inflammatory response by inhibiting the NF-κB pathway (Huang et al., 2019; Jastrząb et al., 2019; Muthumalage & Rahman, 2019). Moreover, recent studies have reported the use of CBD in oral ulcer and chemotherapy-induced OM (Cuba et al., 2020; Qi et al., 2021). However, the *in vivo* application of CBD is limited by its insolubility.

It may be possible to increase the bioavailability of CBD by encapsulating it into a suitable nanoparticle vehicle. Nanomedicine is currently used to treat cancer, fungal infections, biofilm, macular degeneration, and rare genetic diseases (Benoit et al., 2019; Germain et al., 2020; Lammers & Ferrari, 2020). In the case of OM, it may be possible to engineer an effective vehicle by using fucoidan, a hydrophilic natural polysaccharide that has already been applied in tumor-targeting drug delivery (Li et al., 2017; Juenet et al., 2018; Novoyatleva et al., 2019; Jafari et al., 2020; DuRoss et al., 2021). Fucoidan binds strongly to P-selectin, an adhesion molecule that is highly expressed in endothelial and platelet cells under inflammatory conditions and that has been suggested as a potential biomarker in inflammatory response (Juenet et al., 2018; Perkins et al., 2019; Jafari et al., 2020; DuRoss et al., 2021). High levels of P-selectin expression have also been detected in inflammatory endothelial cells of OM (Mafra et al., 2019).

**Scheme 1.**
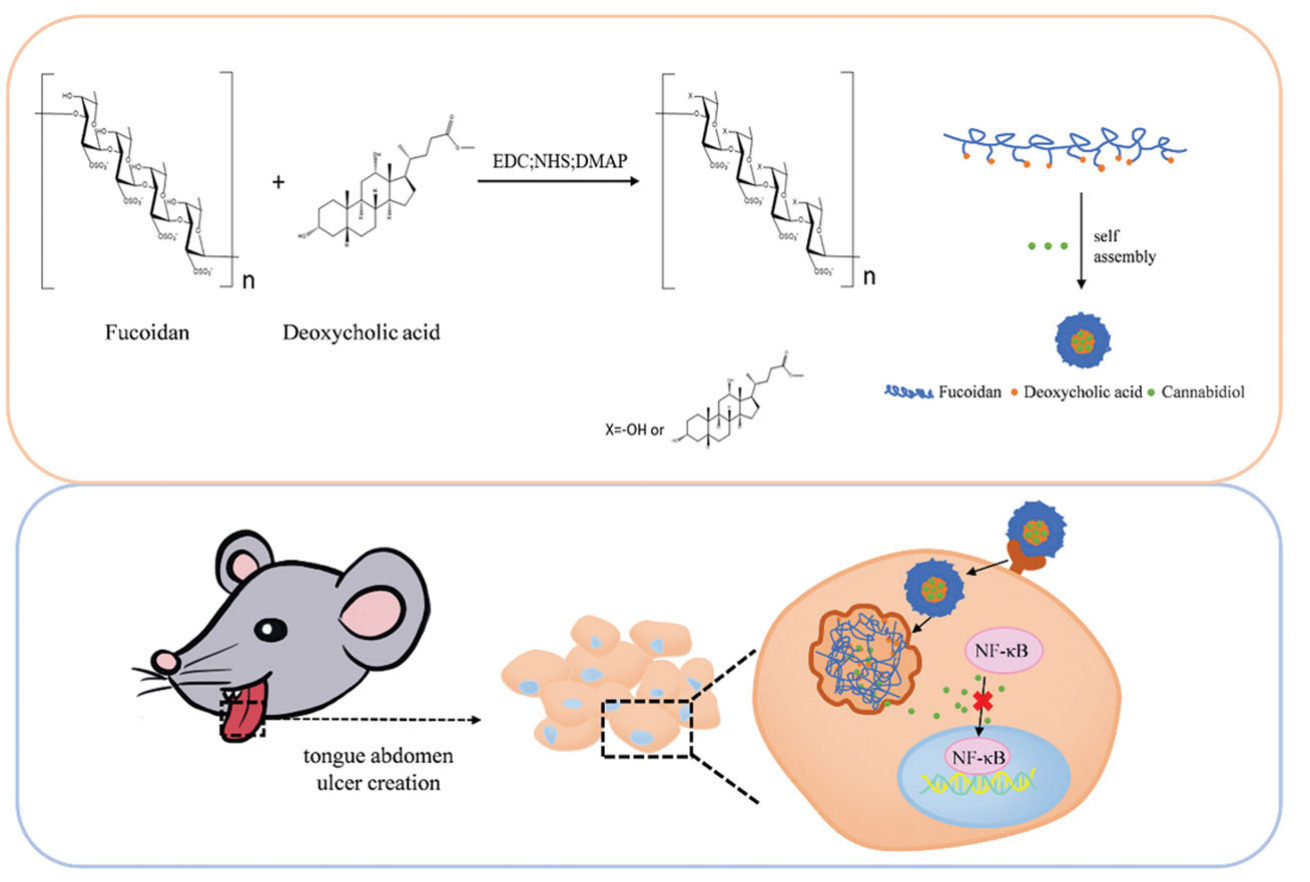
Preparation of cannabidiol/fucoidan–deoxycholic acid nanomicelles and their delivery to inflamed tongue tissue. EDC: *N'*-(Ethylcarbonimidoyl)-*N*,*N*-dimethylpropan-1,3-diamine monohydrochloride; NHS: *N*-hydroxysuccinimide; DMAP: 4-(*N,N*-dimethylamino)pyridine.

Currently, there are only a few studies on the application of nano-drug delivery for OM treatment. Here we developed self-assembled nanomicelles using fucoidan (Fu) and the hydrophobic deoxycholic acid (DOCA) to encapsulate the anti-inflammatory hydrophobic drug CBD (Scheme 1). The resulting Fu–DOCA nanomicelles (hereafter “FD nanomicelles”) not only improved the medicinal properties and therapeutic effect of CBD but also enhanced its accumulation at the inflammation site due to their inflammation-targeting ability. Given the strong affinity of fucoidan for P-selectin, we expect that the CBD-loaded nanomicelles may serve as a new therapeutic approach not only for OM but for other inflammatory diseases as well.

## 2. Materials and methods

### 2.1. Materials

Fucoidan was purchased from Yuanye Co. Ltd. (Shanghai, China). *N'*-(Ethylcarbonimidoyl)- *N*,*N*-dimethylpropan-1,3-diamine monohydrochloride (EDC), 4-(*N,N*-dimethylamino)pyridine (DMAP), and *N*-hydroxysuccinimide (NHS) were purchased from J&K Scientific Co. Ltd. (Beijing, China). Doxorubicin (DOX) was provided by Dalian Meilun Biological Technology Co. Ltd. (Dalian, China). Lipopolysaccharides (LPS) and 5-Fluorouracil (5-Fu) were purchased from Sigma-Aldrich （Shanghai, China）. A defined keratinocyte serum-free medium was purchased from Thermo Fisher Scientific (USA). Mouse anti-CD62P antibody was obtained from Abcam (Cambridge, UK). Cannabidiol was purchased from Yunnan Hempmon Pharmaceutical Co. Ltd.

### 2.2. Synthesis of Fu-DOCA

For the synthesis of Fu-DOCA by esterification, deoxycholate (19.6 mg), EDC (38.4 mg), NHS (23 mg), and DMAP (24.4 mg) were dissolved in 14.25 mL of *N,N*-dimethylformamide (DMF) and stirred at 33 °C for 3.5 h for carboxyl activation. Fucoidan (106 mg) dissolved in 6 mL of DMF at 50 °C was then added dropwise and the reaction mixture was heated at 38 °C for 36 h until it turned faint yellow. An equal amount of water was added to stop the reaction, and the nanomicelles were collected by dialysis and lyophilization. The synthesis of Fu-DOCA was confirmed by ^1^H nuclear magnetic resonance (NMR) and Fourier-transform infrared (FTIR) spectroscopy.

### 2.3. Preparation and characterization of CBD/FD nanomicelles

CBD-loaded FD micelles (hereafter “CBD/FD nanomicelles”) were prepared by a solvent injection method. CBD and FD nanomicelles at a mass ratio of 1:20 were dissolved in methanol and the resulting solution was added dropwise to a tenfold volume of phosphate-buffered saline (PBS). Methanol was then removed by rotary evaporation, and the mean size and zeta potential of the obtained nanomicelles were estimated using a Malvern Zetasizer Nano ZS90 (Malvern Nano ZS, Malvern, UK) instrument. Nanomicelle morphology was observed by transmission electron microscopy (TEM).

To determine their encapsulation efficiency, the CBD/FD nanomicelles were first centrifuged at 10,000 rpm for 10 min to remove unencapsulated CBD. The supernatant was then collected and mixed with the same volume of methanol to disassemble the micelle. The concentration of CBD was determined by high-performance liquid chromatography (HPLC), and the encapsulation efficiency was defined as the ratio of encapsulated to total drug amount.

To evaluate the stability of CBD/FD nanomicelles in serum, 1 mL of nanomicelles was incubated with one volume of fetal bovine serum (FBS) at 37 °C, and the transmittance at 750 nm was measured at 0, 1, 2, 4, 8, 12, and 24 h. A mixture of PBS with FBS was used as a control.

To evaluate the release of CBD *in vitro*, dialysis bags (molecular weight cutoff, 1000 Da) containing 1 mL of CBD/FD nanomicelles or free CBD (CBD: 200 μg/mL) were immersed into 20 mL of 3% sodium dodecyl sulfate-containing PBS and shaken at 80 rpm at 37 °C. Dialysate samples (200 μL) were then collected at 1, 2, 4, 8, 12, 24, and 48 h and replaced by the same amount of fresh release medium. The concentration of CBD was determined by HPLC.

### 2.4. Cell lines and animals

Human oral keratinocytes (HOKs) were cultured at 37 °C in a humidified incubator with 5% CO_2_. C57BL6 mice were purchased from Dossy Experimental Animals Co. Ltd (Chengdu, China). The mice were kept at 15–22 °C and the lighting time was 10–14 h per day. All animal experiments were approved by the Animal Welfare and Ethics Committee of Sichuan University and performed in accordance with the Guidelines for the Care and Use of Laboratory Animals.

### 2.5. Evaluation of inflammation-targeting ability *in vitro*

#### 2.5.1. Inflammation-induced P-selectin expression

When HOKs reached 80–90% confluence in six-well plates, 2 μg/mL LPS was added, followed by incubation for 36 h. The cells were then washed three times with PBS, fixed in 4% polyformaldehyde for 20 min, incubated with 1% Triton X-100 TBS for 30 min, and sealed with Tris-buffered saline solution (TBS) supplemented with 5% FBS for 1.5 h. Afterward, the cells were incubated with anti-CD62p primary antibody at 4 °C overnight. The cells were washed three times with PBS, then incubated with fluorescein isothiocyanate (FITC)-labeled secondary antibody in 5% PBS containing FBS at room temperature for 2 h in the dark. The cells were washed again three times with PBS and the nuclei were labeled upon incubation with 0.5 μg/mL 4′,6-diamidino-2-phenylindole (DAPI) for 5 min in the dark. After washing with TBS, an anti-fluorescence quenching agent was added, and the expression of P-selectin was observed by confocal laser scanning microscopy (LSM800, Carl Zeiss, Germany).

To quantify P-selectin expression, HOKs were cultured in six-well plates overnight and then incubated with 2 μg/mL LPS for 36 h. After digestion, the cells were centrifuged at 2,500 rpm for 3 min. The collected cells were then washed with PBS, fixed, and permeabilized using a fixation/permeabilization kit (BD, Biosciences, USA). Afterward, the cells were incubated for 30 min with anti-CD62P primary antibody diluted at a ratio of 1:100 in PBS containing 1% bovine serum albumin (BSA). After washing three times with PBS, the cells were incubated with FITC-labeled secondary antibody in PBS containing 1% BSA for 30 min in the dark, washed again with PBS, and analyzed by flow cytometry (BD, Biosciences, USA).

#### 2.5.2. Competitive inhibition assay

When HOKs reached 80–90% confluence in six-well plates, they were incubated with 2 μg/mL LPS for 36 h. After the addition of anti-CD62p primary antibody (2 μL/mL) to block cellular receptors, the HOKs were treated for 4 h with DOX-loaded FD nanomicelles (DOX/FD) in which the concentration of DOX was 0.5 μg/mL, digested with trypsin, washed with PBS, and measured by flow cytometry. HOKs without receptor blocking were used as control.

#### 2.5.3. In vitro anti-inflammation assay

In vitro anti-inflammatory assay was done to verify the anti-inflammatory effects of CBD/FD. HOKs were cultured in 12-well plates, and when the cells reached 80–90% confluence, they were incubated with 2 μg/mL LPS for 36 hours. Then HOKs were incubated with anti-CD62P primary antibody at a concentration of 2 μL/mL for 2 h. After replacing the medium, cells were incubated with CBD/FD (CBD, 15 μg/mL) for 4 h. The cells were again stimulated with 2 μg/mL LPS for 36 h after the medium was replaced, then inflammatory factors IL-1β and TNF-α in the cell culture medium were detected by ELISA.

### 2.6. Om mouse model

To establish the *in vivo* OM model, C57BL6 mice were intraperitoneally injected with 50 mg/kg 5-Fu (Im et al., 2019). Then a rounded filter paper with a diameter of 3 mm was immersed in 50% acetic acid and placed for 2 min on the front one-third of the tongue abdomen of mice anesthetized with 4% chloral hydrate (Nodai et al., 2018).

### 2.7. Evaluation of inflammation-targeting ability *in vivo*

*In vivo* inflammation-targeted ability of FD was evaluated by two administration pathways including intravenous injection and in situ dripping. Eight mice were randomly divided into two groups by random number sequence (*n* = 4 per group). Free DOX or DOX/FD (DOX: 2 mg/mg), which were prepared similar to CBD/FD, were then injected *via* the tail vein at 36 h after ulcer creation. The mouse tongues were collected at 4 and 12 h post-administration.

For *in situ* dripping, 18 mice were randomly divided into two groups (*n* = 9 per group) at 36 h after ulcer creation. Free DOX or DOX/FD nanomicelles (30 μL, DOX: 0.2 mg/mL) were then dripped onto the tongue ulcer. After administration, the mice have fasted for 1 h and their tongues were collected at 2, 4, and 12 h post-administration. Fluorescent images of the collected samples were obtained using an *in vivo* imaging spectrum system (Caliper Life Sciences, USA). The samples collected at 4 h were then fixed in 4% paraformaldehyde and processed for immunofluorescence as described in section Inflammation-induced P-selectin expression.

### 2.8. *In situ* OM treatment assay

The therapeutic effect of CBD/FD was evaluated by intravenous injection or *in situ* dripping. The investigator who performed the assay was unaware of the group assignment. The day of ulcer creation was considered as day 0. Mice without obvious tongue inflammation one day after ulcer creation would be excluded. On days 1 and 3, 30 μL of free CBD or CBD/FD nanomicelles (CBD: 1 mg/mL) was dripped onto the inflamed tongue region, and the mice have fasted for 1 h. Each day after ulcer creation, the ulcer area was observed. On days 2 and 4, the mouse tongues were collected and fixed in 4% polyformaldehyde. Hematoxylin and eosin (H&E) staining was used to assess the size of the open ulcer and tissue healing, while the infiltration of neutrophil or marrow-derived suppressor cells (Ly6G) and the nuclear localization of NF-κB in the inflamed region was observed by immunohistochemistry. The same process using 0.2 mL of free CBD or CBD/FD nanomicelles (CBD: 0.5 mg/mL) was applied to evaluate the therapeutic effect of CBD/FD at 1 and 3 days after intravenous administration through the tail vein.

### 2.9. Statistical analysis

The two-tailed *t*-test and one-way ANOVA test were used for 2 groups and multiple groups statistical comparisons respectively. Differences associated with *P < .05, **P < .01, and ***P < .001 were considered statistically significant.

## 3. Results and discussion

### 3.1. Synthesis of Fu-DOCA

The synthesis of Fu-DOCA was confirmed by ^1^H nuclear magnetic resonance (^1^H NMR) and Fourier-transform infrared (FTIR) spectroscopy. As shown in Supplementary Figure S1, the characteristic peaks of free fucoidan and the carboxyl peak of free DOCA were detected at 3.5–4.0 and 11.9 ppm, respectively. After esterification, the carboxyl peak disappeared, indicating the successful binding of DOCA to fucoidan. In addition, the absorption peak of DOCA at 3550 cm^−1^ corresponding to the carboxyl group disappeared in the FTIR spectrum of FD, while new peaks were detected at 1711, 1090, and 1040 cm^−1^, further confirming the ester bond formation (Supplementary Figure S2).

### 3.2. Characterization of CBD/FD nanomicelles

**Figure 1.**
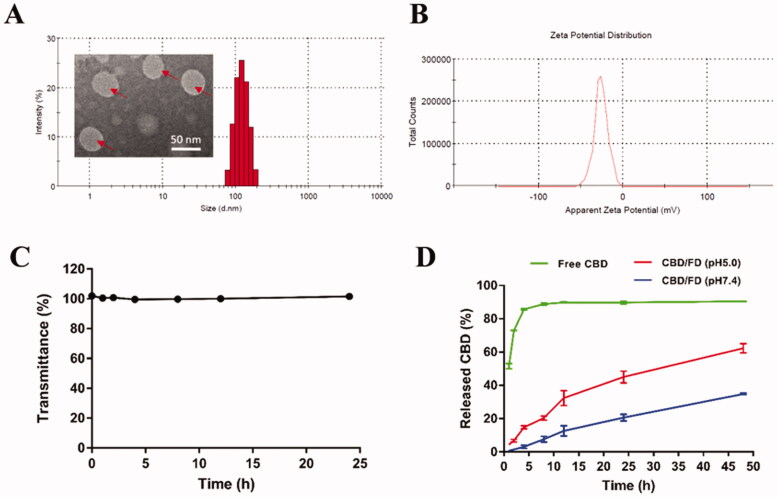
Characterization of nanomicelles. (A) Size distribution, (B) zeta potential, and (C) transmittance of CBD/FD nanomicelles. (D) Drug release profile under different pH conditions. CBD: cannabidiol; FD: fucoidan.

The average size of CBD/FD nanomicelles was 118.75 ± 1.46 nm, with a polydispersity index of 0.0315 ± 0.0265 (Figure 1(A)). The encapsulation efficiency of CBD into FD nanomicelles was 88.8 ± 0.2%, while the zeta potential of the prepared nanomicelles was estimated at −24.45 ± 1.25 mV (Figure 1(B)). Moreover, a transmittance of CBD/FD nanomicelles remained above 90% for 24 h with no apparent protein aggregation or adsorption, indicating good serum stability (Figure 1(C)). CBD/FD nanomicelles exhibited a sustained-release profile at pH 7.4, indicating greater drug stability in systemic circulation than free CBD which was rapidly released (Figure 1(D)). Interestingly, the release of CBD was faster under lysosomal pH conditions (5.0) due to ester bond hydrolysis, suggesting that CBD would be efficiently released after the internalization of CBD/FD nanomicelles into cells. This has been similarly reported in previous studies (Wei et al., 2013; Long et al., 2018). Actually, it is speculated that the esterase abundant in lysosomes would facilitate drug release more forcefully.

### 3.3. *In vitro* inflammation-targeting and anti-inflammation ability of FD micelles

**Figure 2.**
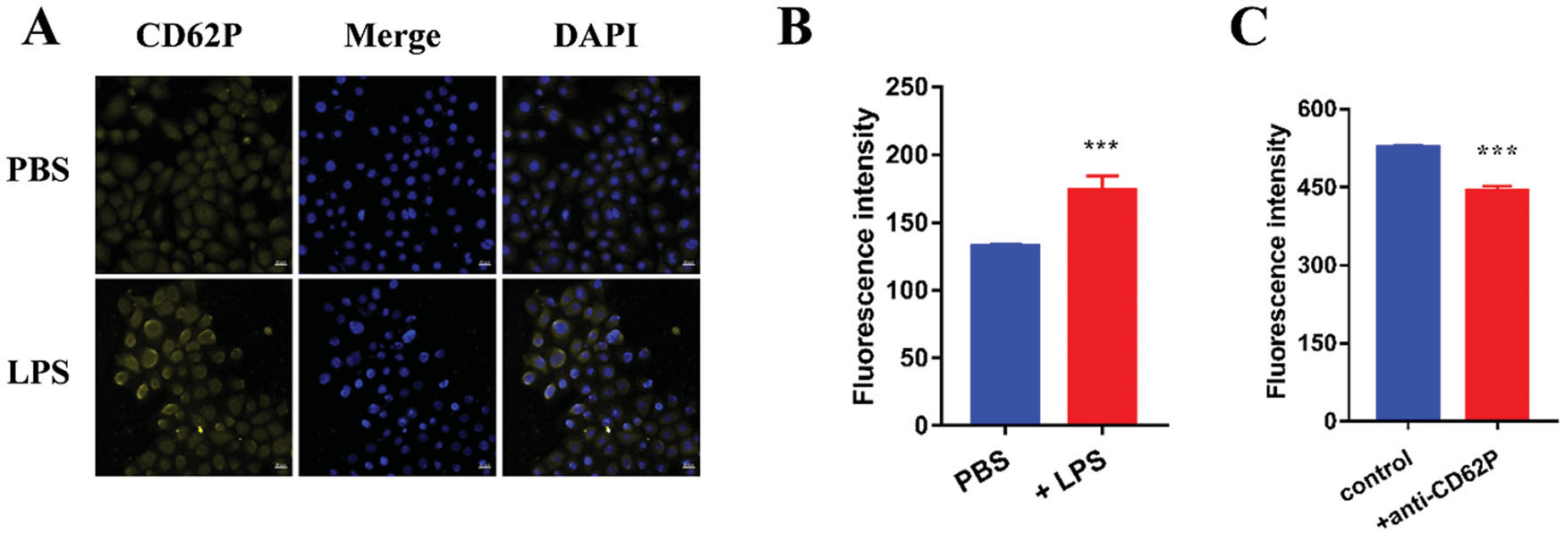
(A,B) LPS-induced P-selectin expression determined by (A) confocal microscopy (scale bar, 20 μm) and (B) flow cytometry (*n* = 3). (C) Anti-CD62p antibody inhibited the uptake of FD micelles in LPS-induced HOKs (*n* = 3). Data are shown as mean ± SD. CBD: cannabidiol; DAPI: 4',6-diamidino-2-phenylindole; LPS: lipopolysaccharide; PBS: phosphate-buffered saline.

Given that fucoidan show a high affinity for P-selectin (Shamay et al., 2016; Mafra et al., 2019; Perkins et al., 2019), the expression of P-selectin in FD-treated cells was assessed by confocal microscopy and flow cytometry. Stronger fluorescence was observed after incubation with 2 μg/mL LPS (Figure 2(A)). Moreover, treatment of HOKs with 2 μg/mL LPS significantly increased the expression of P-selectin (Figure 2(B)), suggesting that inflammation can induce P-selectin expression in oral keratinocytes.

A competitive inhibition assay was performed to further confirm the role of P-selectin in the cellular uptake of FD nanomicelles. The expression of P-selectin in HOKs was blocked with the anti-CD62P antibody. The cellular uptake of DOX/FD nanomicelles was significantly inhibited (Figure 2(C)), suggesting that P-selectin mediates the ability of FD nanomicelles to target inflammation.

The secretion of inflammatory factors IL-1β and TNF-α were detected. As shown in Supplementary Figure S5, LPS induced the secretion of inflammatory factors TNF-α and IL-1β by HOK cells, and the level of inflammatory factors were significantly reduced by CBD/FD. Pretreatment with anti-CD62p primary antibody attenuated the anti-inflammatory effect of CBD/FD as antibody treatment inhibited internalization of CBD/FD. The result was consistent with competitive inhibition assay, and again demonstrated the role of enhanced P-selectin expression in CBD/FD internalization induced by inflammation.

### 3.4. *In vivo* inflammation-targeting ability of FD nanomicelles

**Figure 3.**
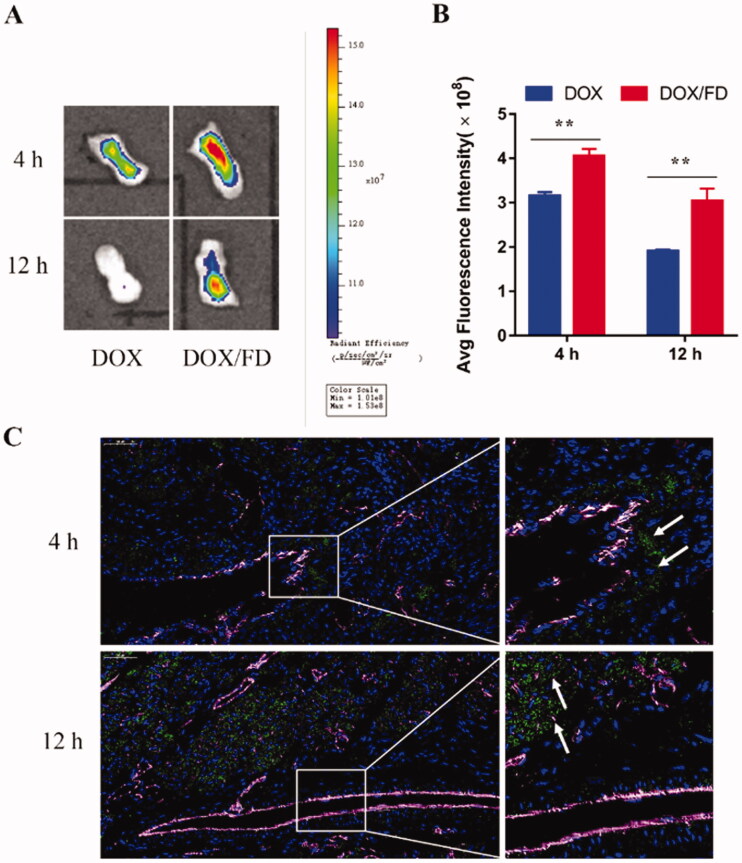
*In vivo* inflammation-targeting ability of fucoidan–deoxycholic acid (FD) nanomicelles after intravenous injection *via* the tail vein. (A) Fluorescence images of the tongue were obtained using an *in vivo* imaging spectrum system. (B) Semi-quantitative analysis of fluorescence images (*n* = 3). Data are shown as mean ± SD. (C) Immunofluorescence images of the tongue after administration of DOX/FD nanomicelles (blue, nucleus; green, DOX/FD nanomicelles; purple, blood vessels). DOX: doxorubicin. Scale bar, 50 μm.

The inflammation-targeting ability of FD nanomicelles was evaluated *in vivo* after intravenous injection or *in situ* dripping of DOX/FD nanomicelles. Compared to free DOX, DOX/FD nanomicelles showed better targeting and retention ability at 4 and 12 h after intravenous administration (Figures 3(A,B)), probably due to the longer half-life of the drug and the inflammation-targeting ability of the nanomicelles. Moreover, as shown in Figure 3(C), at 4 h post-administration, the DOX/FD nanomicelles were distributed around the vessels, while at 12 h after administration, they were detected inside the tissue, indicating the good retention of FD nanomicelles (and therefore of the drug payload) in the inflamed tongue tissue.

**Figure 4.**
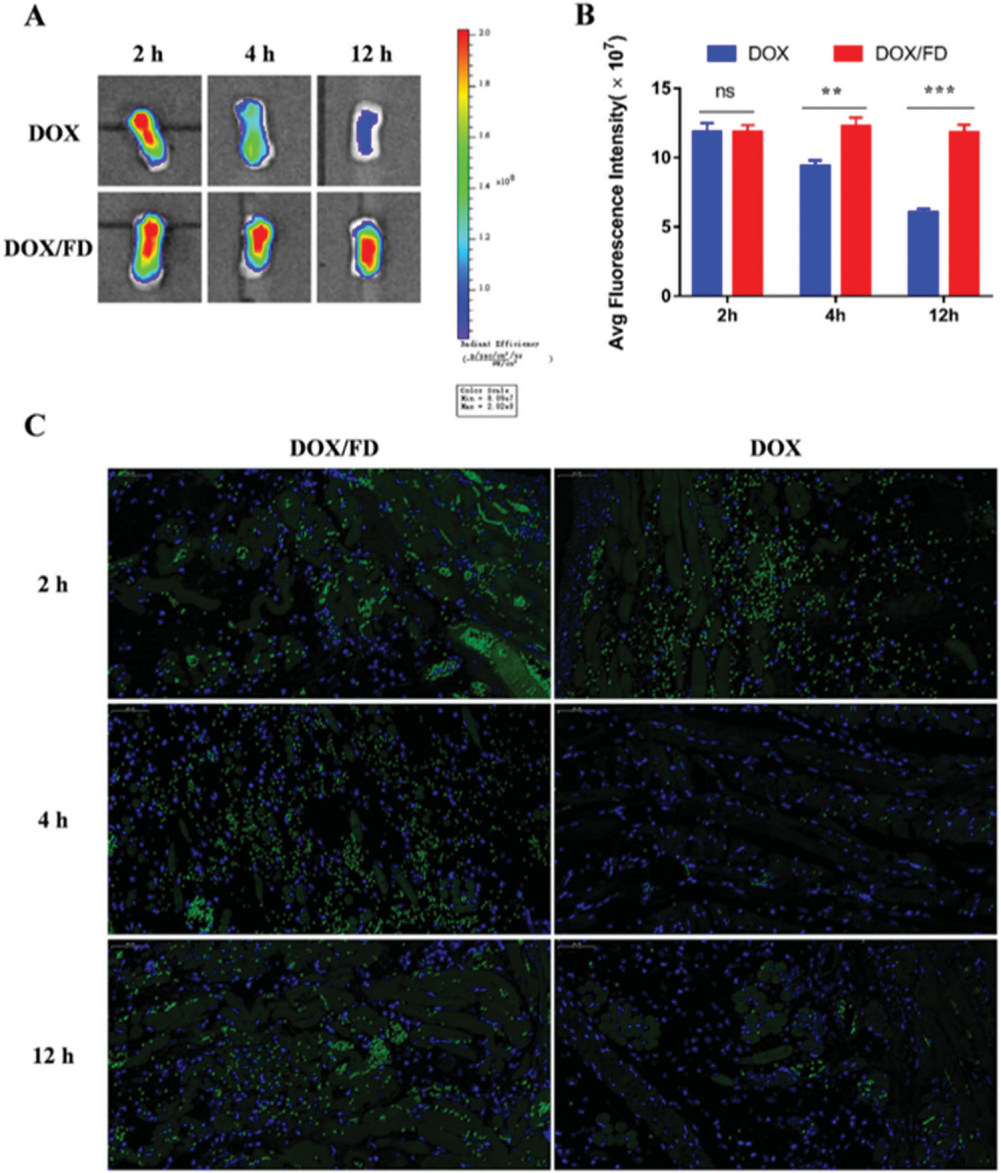
*In vivo* inflammation-targeting ability of fucoidan–deoxycholic acid (FD) nanomicelles after *in situ* dripping. (A) Fluorescence images of the tongue were obtained using an *in vivo* imaging spectrum system. (B) Semi-quantitative analysis of fluorescence images (*n* = 3). Data are shown as mean ± SD. (C) Immunofluorescence images of tongues after administration of DOX/FD nanomicelles (blue, nucleus; green, DOX/FD nanomicelles; purple, blood vessels). DOX: doxorubicin. Scale bar, 50 μm.

In contrast to intravenous administration, free DOX and DOX/FD nanomicelles showed similar retention in the tongue tissue at 2 h after *in situ* dripping. However, the retention of free DOX decreased over time, while that of DOX/FD nanomicelles remained almost unchanged even by 12 h post-administration, suggesting that FD can significantly increase drug retention in inflamed tissues (Figures 4(A,B)). Immunofluorescence imaging also showed that the accumulation of DOX/FD nanomicelles in the tongue tissue was significantly higher than that of free DOX (Figure 4(C)). Thus, although these inflammation-targeting nanocarriers may not be cleared away as easily as free drugs, they can be retained efficiently at the target site due to the high affinity of FD for P-selectin.

### 3.5. *In situ* OM treatment

**Figure 5.**
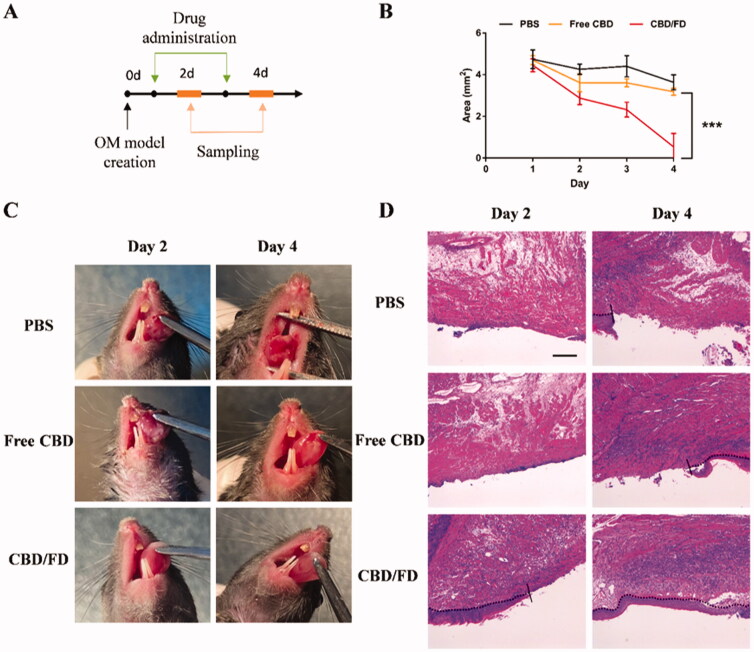
The therapeutic effect of CBD/FD nanomicelles on tongue ulcers after intravenous administration. (A) Drug administration and sampling. (B) Change in the ulcer area over time (*n* = 5). Data are shown as mean ± SD. (C) Tongue ulcer images at 2 and 4 days post-administration. (D) Hematoxylin and eosin images of tongue ulcers. Solid lines indicate the ulcer boundary, and dotted lines indicate the epithelial-stromal boundary. Scale bar, 100 μm.

The therapeutic effect of the CBD/FD nanomicelles on the tongue ulcer was evaluated by observing the ulcer area every day after intravenous injection or *in situ* dripping of free CBD or CBD/FD nanomicelles (Figures 5(A),7(A)). After intravenous administration, CBD/FD nanomicelles led to significantly lower ulceration degree (Figure 5(C)) and ulcer area (Figure 5(B)) than PBS or free CBD, due to the inflammation-targeting and high-retention properties of the FD nanomicelles. These results were consistent with those obtained by H&E staining (Figure 5(D)).

**Figure 6.**
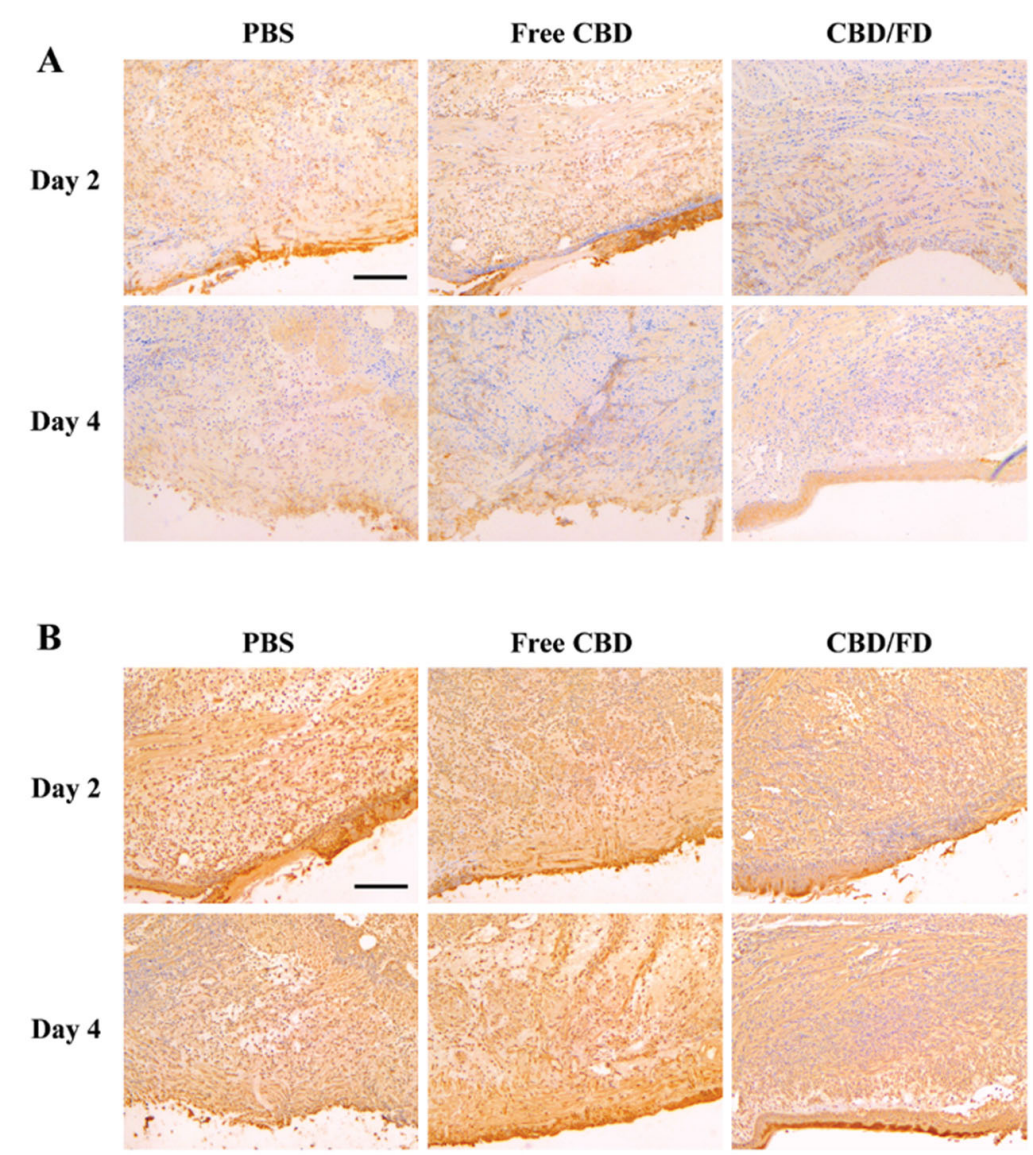
Immunohistochemical staining of (A) Ly6G cells and (B) NF-κB p65 in tongue ulcers after intravenous administration of CBD/FD nanomicelles. Scale bar, 100 μm.^+^

To further evaluate the inflammation degree, the presence of Ly6G cells, which include polymorphonuclear neutrophils or polymorphonuclear myeloid-derived suppressor cells (MDSCs), was assessed by Ly6G staining. Normal tongue tissue served as control, where no Ly6G cell infiltration was observed (Supplementary Figure S5). Compared to PBS and free CBD, CBD/FD nanomicelles significantly reduced Ly6G cell infiltration, exhibiting an improved anti-inflammatory effect. (Figure 6(A)). The interaction of P-selectin glycoprotein ligand 1 (PSGL-1) expressed on Ly6G + cells and P-selectin expressed on vascular endothelial cells mediated the process of cell infiltration. Therefore, we speculate that the reduced infiltration of Ly6G cells is partly due to the ability of fucoidan competitively bind to P-selectin. It is analogous to how low-molecular-weight heparin can competitively bind P-selectin and thereby inhibits the recruitment of MDSCs (Stadtmann et al., 2013; Long et al., 2020).

Since CBD can inhibit the nuclear transcription of NF-κB (Huang et al., 2019; Jastrząb et al., 2019; Muthumalage & Rahman, 2019), we also performed immunostaining of tongue ulcers for NF-κB p65 and observed its nuclear localization. On day 2, the nucleus in the PBS and free CBD groups was clearly stained, suggesting the active nuclear transcription of NF-κB. In contrast, the nuclear transcription of NF-κB was inhibited after the first administration of CBD/FD nanomicelles (Figure 6(B)). These results clearly support that the CBD/FD micelles show promising anti-inflammatory and healing effects.

**Figure 7.**
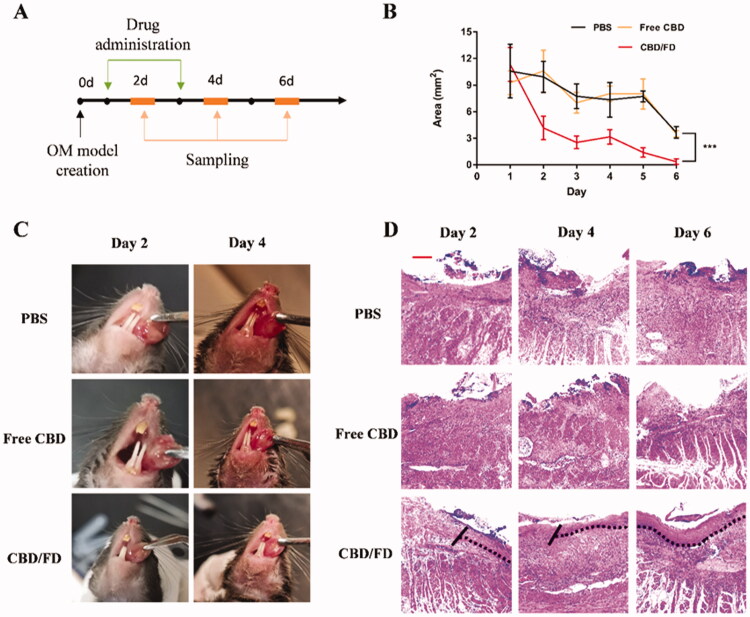
The therapeutic effect of CBD/FD nanomicelles on tongue ulcers after *in situ* dripping. (A) Drug administration and sampling. (B) Change in the ulcer area over time (*n* = 5). Data are shown as mean ± SD. (C) Tongue ulcer images at 2 and 4 days post-administration. (D) Hematoxylin and eosin images of tongue ulcers. Solid lines indicate the ulcer boundary, and dotted lines indicate the epithelial-stromal boundary. Scale bar, 100 μm.

**Figure 8.**
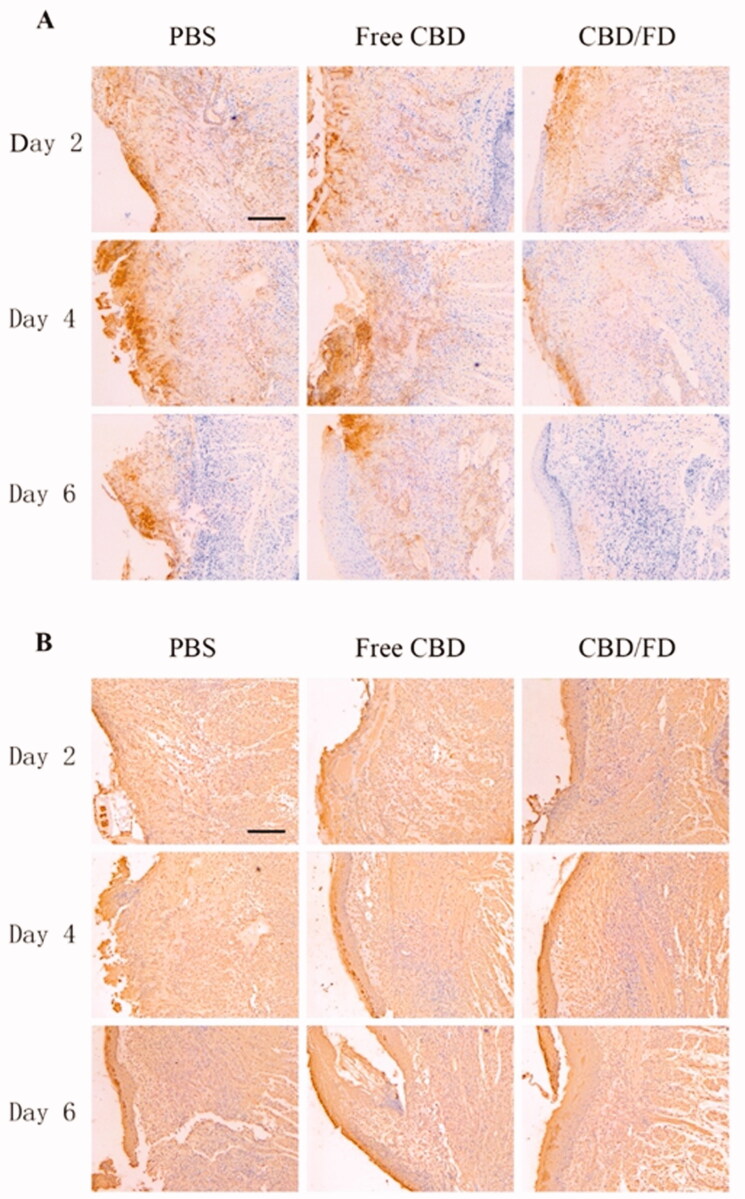
Immunohistochemical staining of (A) Ly6G cells and (B) NF-κB p65 in tongue ulcers after *in situ* dripping of CBD/FD nanomicelles. Scale bar, 100 μm.^+^

The same method was used to evaluate the therapeutic effect of CBD/FD nanomicelles administered by *in situ* dripping. As expected, CBD/FD nanomicelles showed excellent healing and anti-inflammatory effects (Figures 7–8), which was attributed to the enhanced drug accumulation at the site of inflammation.

## 4. Conclusion

Few studies have examined treatments for OM, especially those involving nanomedicine. In this study, inspired by the use of inflammation-targeted nanomedicine for cancer treatment, we prepared CBD-loaded inflammation-targeting nanomicelles for the treatment of OM. The CBD/FD nanomicelles exhibited excellent inflammation-targeting ability and showed outstanding anti-inflammatory and healing effects after either *in situ* dripping or intravenous administration. These results suggest the great potential of CBD/FD nanomicelles for clinical application.

## Supplementary Material

Supplemental Material

## Data Availability

Not applicable

## References

[CIT0001] Atalay S, Jarocka-Karpowicz I, Skrzydlewska E. (2019). Antioxidative and anti-inflammatory properties of cannabidiol. Antioxidants 9:21. 10.3390/antiox9010021 PMC7023045 31881765

[CIT0002] Benoit DSW, Sims KR, Fraser D. (2019). Nanoparticles for oral biofilm treatments. ACS Nano 13:4869–75. 31033283 10.1021/acsnano.9b02816 PMC6707515

[CIT0003] Blakaj A, Bonomi M, Gamez ME, Blakaj DM. (2019). Oral mucositis in head and neck cancer: evidence-based management and review of clinical trial data. Oral Oncol 95:29–34. 31345391 10.1016/j.oraloncology.2019.05.013

[CIT0004] Burstein S. (2015). Cannabidiol (cbd) and its analogs: a review of their effects on inflammation. Bioorg Med Chem 23:1377–85. 25703248 10.1016/j.bmc.2015.01.059

[CIT0005] Cuba LF, Salum FG, Guimarães FS, et al. (2020). Cannabidiol on 5-fu-induced oral mucositis in mice. Oral Dis 26:1483–93. 32400905 10.1111/odi.13413

[CIT0006] Dos-Santos-Pereira M, Guimarães FS, Del-Bel E, et al. (2020). Cannabidiol prevents lps-induced microglial inflammation by inhibiting ros/nf-κb-dependent signaling and glucose consumption. Glia 68:561–73. 31647138 10.1002/glia.23738

[CIT0007] DuRoss AN, Landry MR, Thomas CR, et al. (2021). Fucoidan-coated nanoparticles target radiation-induced p-selectin to enhance chemoradiotherapy in murine colorectal cancer. Cancer Lett 500:208–19. 33232787 10.1016/j.canlet.2020.11.021 PMC9392493

[CIT0008] Elad S, Cheng KKF, Lalla RV, et al. (2020). Mascc/isoo clinical practice guidelines for the management of mucositis secondary to cancer therapy. Cancer 126:4423–31. 32786044 10.1002/cncr.33100 PMC7540329

[CIT0009] Elad S, Yarom N. (2019). The search for an effective therapy and pain relief for oral mucositis. JAMA 321:1459–61. 30990535 10.1001/jama.2019.3269

[CIT0010] Germain M, Caputo F, Metcalfe S, et al. (2020). Delivering the power of nanomedicine to patients today. J Control Release 326:164–71. 32681950 10.1016/j.jconrel.2020.07.007 PMC7362824

[CIT0011] Huang Y, Wan T, Pang N, et al. (2019). Cannabidiol protects livers against nonalcoholic steatohepatitis induced by high-fat high cholesterol diet via regulating NF-κB and NLRP3 inflammasome pathway. J Cell Physiol 234:21224–34. 31032942 10.1002/jcp.28728

[CIT0012] Im K-I, Nam Y-S, Kim N, et al. (2019). Regulation of hmgb1 release protects chemoradiotherapy-associated mucositis. Mucosal Immunol 12:1070–81. 30647411 10.1038/s41385-019-0132-x

[CIT0013] Jafari M, Sriram V, Xu Z, et al. (2020). Fucoidan-doxorubicin nanoparticles targeting p-selectin for effective breast cancer therapy. Carbohydr Polym 249:116837. 32933681 10.1016/j.carbpol.2020.116837

[CIT0014] Jastrząb A, Gęgotek A, Skrzydlewska E. (2019). Cannabidiol regulates the expression of keratinocyte proteins involved in the inflammation process through transcriptional regulation. Cells 8(8):827. 10.3390/cells8080827 PMC6721680 31382646

[CIT0015] Juenet M, Aid-Launais R, Li B, et al. (2018). Thrombolytic therapy based on fucoidan-functionalized polymer nanoparticles targeting p-selectin. Biomaterials 156:204–16. 29216534 10.1016/j.biomaterials.2017.11.047

[CIT0016] Lammers T, Ferrari M. (2020). The success of nanomedicine. Nano Today 31:100853. 34707725 10.1016/j.nantod.2020.100853 PMC7611893

[CIT0017] Li B, Juenet M, Aid-Launais R, et al. (2017). Development of polymer microcapsules functionalized with fucoidan to target p-selectin overexpressed in cardiovascular diseases. Adv Healthc Mater 6(4):27943662. 10.1002/adhm.201601200 27943662

[CIT0018] Long Y, Lu Z, Mei L, et al. (2018). Enhanced melanoma-targeted therapy by "fru-blocked" phenyboronic acid-modified multiphase antimetastatic micellar nanoparticles. Adv Sci 5:1800229. 10.1002/advs.201800229 PMC6247072 30479911

[CIT0019] Long Y, Lu Z, Xu S, et al. (2020). Self-delivery micellar nanoparticles prevent premetastatic niche formation by interfering with the early recruitment and vascular destruction of granulocytic myeloid-derived suppressor cells. Nano Lett 20:2219–29. 31823615 10.1021/acs.nanolett.9b03883

[CIT0020] Luo J, Bian L, Blevins MA, et al. (2019). Smad7 promotes healing of radiotherapy-induced oral mucositis without compromising oral cancer therapy in a xenograft mouse model. Clin Cancer Res 25:808–18. 30185419 10.1158/1078-0432.CCR-18-1081 PMC6335168

[CIT0021] Mafra C, Vasconcelos RC, de Medeiros CACX, et al. (2019). Gliclazide prevents 5-fu-induced oral mucositis by reducing oxidative stress, inflammation, and p-selectin adhesion molecules. Front Physiol 10:327. 30971955 10.3389/fphys.2019.00327 PMC6445135

[CIT0022] Muthumalage T, Rahman I. (2019). Cannabidiol differentially regulates basal and lps-induced inflammatory responses in macrophages, lung epithelial cells, and fibroblasts. Toxicol Appl Pharmacol 382:114713. 31437494 10.1016/j.taap.2019.114713 PMC6917034

[CIT0023] Nodai T, Hitomi S, Ono K, et al. (2018). Endothelin-1 elicits trp-mediated pain in an acid-induced oral ulcer model. J Dent Res 97:901–8. 29518348 10.1177/0022034518762381

[CIT0024] Novoyatleva T, Kojonazarov B, Owczarek A, et al. (2019). Evidence for the fucoidan/p-selectin axis as a therapeutic target in hypoxia-induced pulmonary hypertension. Am J Respir Crit Care Med 199:1407–20. 30557519 10.1164/rccm.201806-1170OC

[CIT0025] Pacher P, Kogan NM, Mechoulam R. (2020). Beyond thc and endocannabinoids. Annu Rev Pharmacol Toxicol 60:637–59. 31580774 10.1146/annurev-pharmtox-010818-021441

[CIT0026] Perkins LA, Anderson CJ, Novelli EM. (2019). Targeting p-selectin adhesion molecule in molecular imaging: P-selectin expression as a valuable imaging biomarker of inflammation in cardiovascular disease. J Nucl Med 60:1691–7. 31601694 10.2967/jnumed.118.225169 PMC6894375

[CIT0027] Pisanti S, Malfitano AM, Ciaglia E, Lamberti A, et al. (2017). Cannabidiol: state of the art and new challenges for therapeutic applications. Pharmacol Ther 175:133–50. 28232276 10.1016/j.pharmthera.2017.02.041

[CIT0028] Pulito C, Cristaudo A, Porta CL, et al. (2020). Oral mucositis: the hidden side of cancer therapy. J Exp Clin Cancer Res 39:210. 33028357 10.1186/s13046-020-01715-7 PMC7542970

[CIT0029] Qi X, Lin W, Wu Y, et al. (2021). Cbd promotes oral ulcer healing via inhibiting cmpk2-mediated inflammasome. J Dent Res 101(2):206–15. 10.1177/00220345211024528 34269108

[CIT0030] Riley P, McCabe MG, Glenny A-M. (2016). Oral cryotherapy for preventing oral mucositis in patients receiving cancer treatment. JAMA Oncol 2:1365–6. 27583808 10.1001/jamaoncol.2016.2680

[CIT0031] Shamay Y, Elkabets M, Li H, et al. (2016). P-selectin is a nanotherapeutic delivery target in the tumor microenvironment. Sci Transl Med 8:345ra387. 10.1126/scitranslmed.aaf7374 PMC5064151 27358497

[CIT0032] Sio TT, Le-Rademacher JG, Leenstra JL, et al. (2019). Effect of doxepin mouthwash or diphenhydramine-lidocaine-antacid mouthwash vs placebo on radiotherapy-related oral mucositis pain: the alliance a221304 randomized clinical trial. JAMA 321:1481–90. 30990550 10.1001/jama.2019.3504 PMC6484809

[CIT0033] Spielberger R, Stiff P, Bensinger W, et al. (2004). Palifermin for oral mucositis after intensive therapy for hematologic cancers. N Engl J Med 351:2590–8. 15602019 10.1056/NEJMoa040125

[CIT0034] Stadtmann A, Germena G, Block H, et al. (2013). The psgl-1-l-selectin signaling complex regulates neutrophil adhesion under flow. J Exp Med 210:2171–80. 24127491 10.1084/jem.20130664 PMC3804951

[CIT0035] Wei H, Zhuo R-X, Zhang X-Z. (2013). Design and development of polymeric micelles with cleavable links for intracellular drug delivery. JPiPS 38:503–35.

