# Supplementary material for: Inflammation-targeted cannabidiol-loaded nanomicelles for enhanced oral mucositis treatment
Source: Drug Deliv. 2022 Apr 25;29(1):1272–81. doi: 10.1080/10717544.2022.2027572 (PMC9045765; doi:10.1080/10717544.2022.2027572)
Supplement: Supplemental Material [file IDRD_A_2027572_SM8205.docx]

**Inflammation-targeted cannabidiol-loaded nanomicelles for enhanced oral mucositis treatment**

Yingke Liu^a^, Xingying Qi^a^, Yashi Wang^b^, Man Li^b^, Quan Yuan^a*^, Zhihe Zhao^a*^

a State Key Laboratory of Oral Diseases, National Clinical Research Center for Oral Diseases, West China Hospital of Stomatology, Sichuan University, Chengdu, China

b Key Laboratory of Drug-Targeting and Drug Delivery System of the Education Ministry and Sichuan Province, Sichuan Engineering Laboratory for Plant-Sourced Drug and Sichuan Research Center for Drug Precision Industrial Technology, Sichuan University, Chengdu, China

*Corresponding Author: Zhihe Zhao, Quan Yuan

Email: [zhzhao@scu.edu.cn](mailto:zhzhao@scu.edu.cn) (Z. Zhao), [yuanquan@scu.edu.cn](mailto:yuanquan@scu.edu.cn) (Q. Yuan)

Tel/Fax:+86 28 85501435 (Z. Zhao), +86 28 85501441 (Q. Yuan)


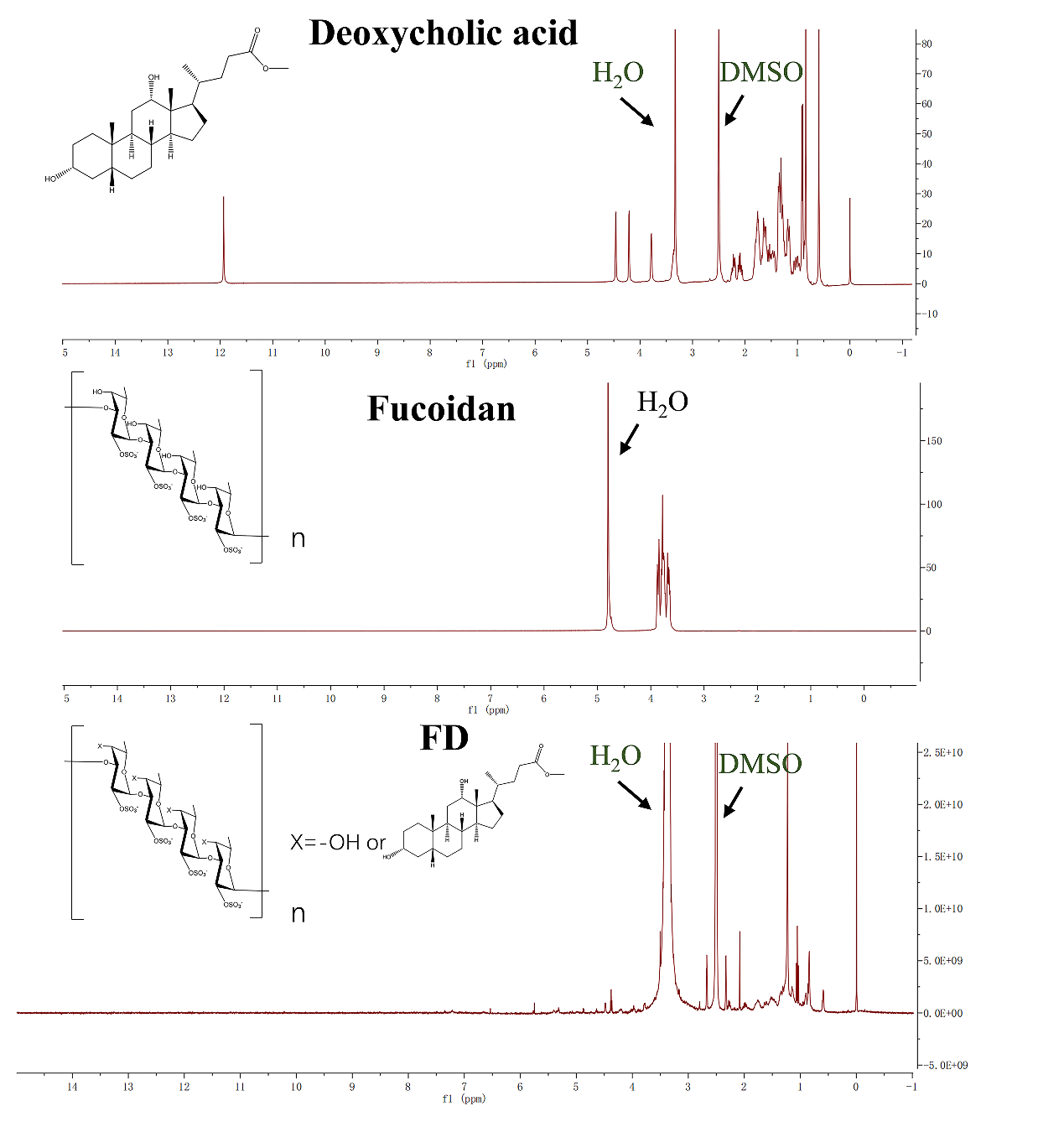


Supplementary Figure S1. ^1^H NMR of fucoidan, deoxycholic acid and FD.


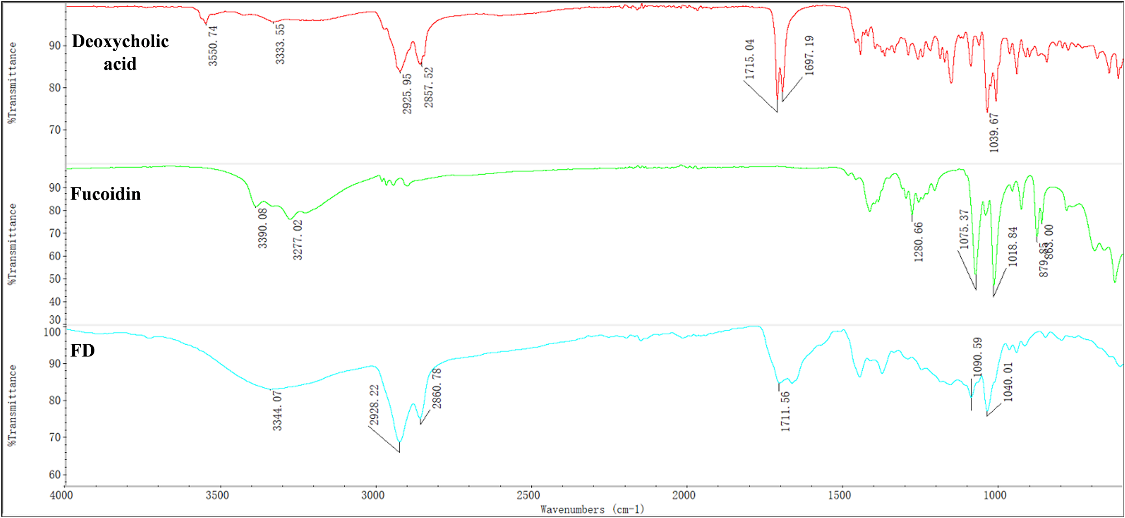


Supplementary Figure S2. FI-TR spectrograms of fucoidan, deoxycholic acid and FD.


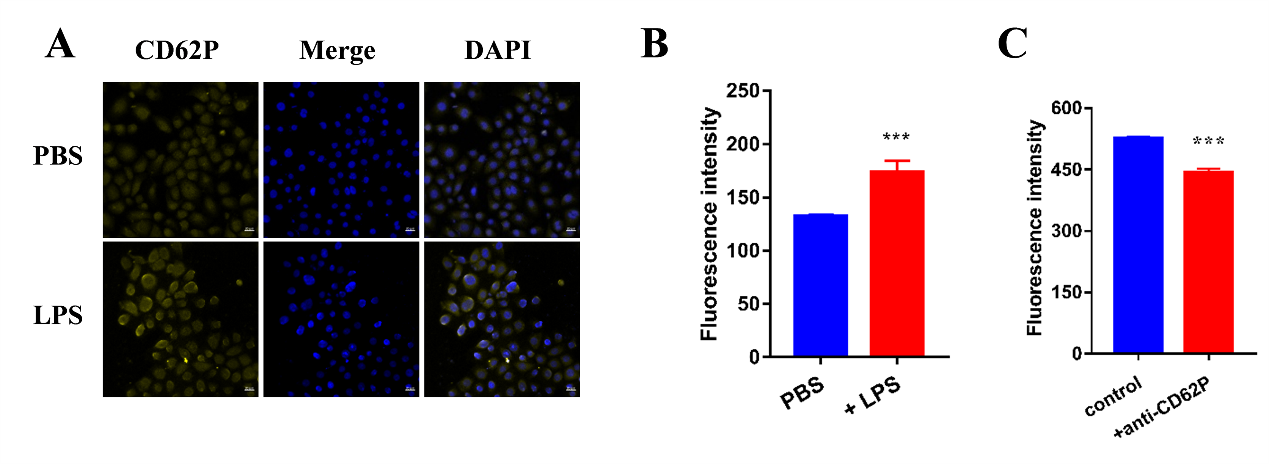


Supplementary Figure S3. (A,B) LPS-induced P-selectin expression determined by (A) confocal microscopy (scale bar, 20 μm) and (B) flow cytometry (n = 3). (C) Anti-CD62p antibody inhibited the uptake of FD micelles in LPS-induced HOKs (n = 3). Data are shown as mean ± SD. CBD: cannabidiol; DAPI: 4',6-diamidino-2-phenylindole; LPS: lipopolysaccharide; PBS: phosphate-buffered saline.


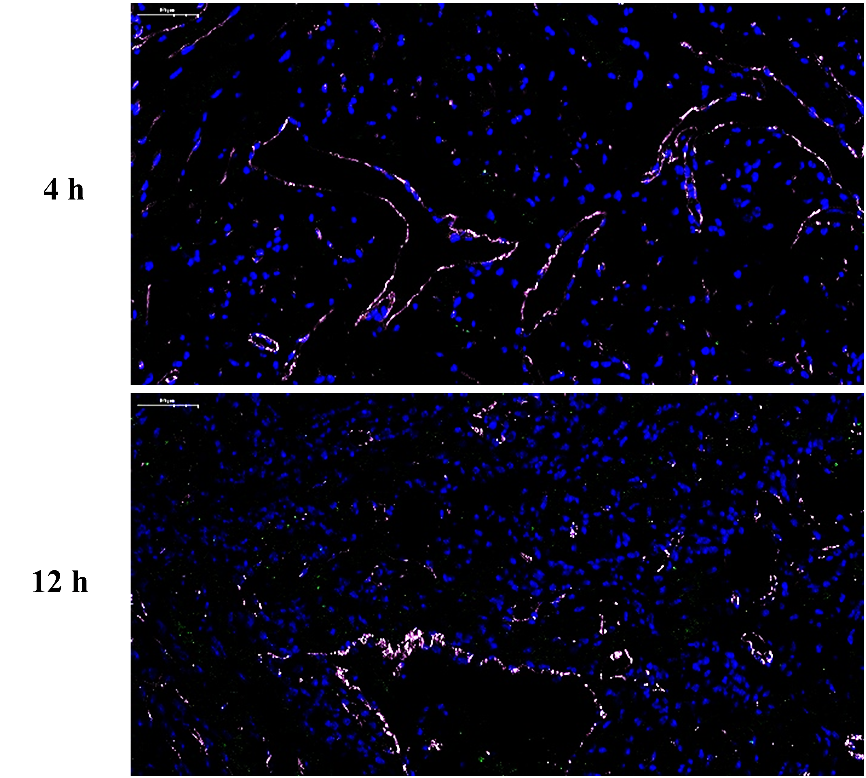


Supplementary Figure S4. In-vivo inflammation-targeted evaluation by intravenous administration. Immunofluorescence images of tongues after free DOX administration. Blue, Nucleus. Green, DOX. Purple, Blood vessel. Scale bar, 50 μm.


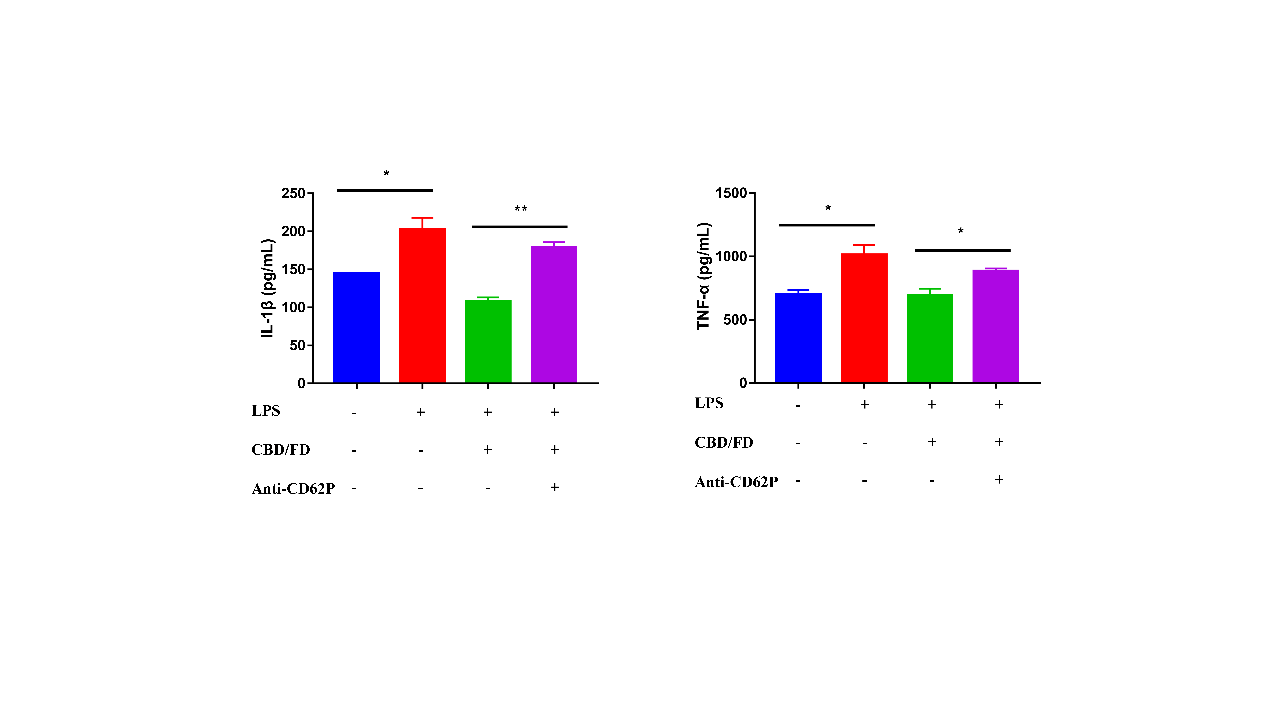


Supplementary Figure S5. The level of inflammatory factors TNF-α and IL-1β secreted by HOK cells after treatment of LPS, CBD/FD and Anti-CD62P.


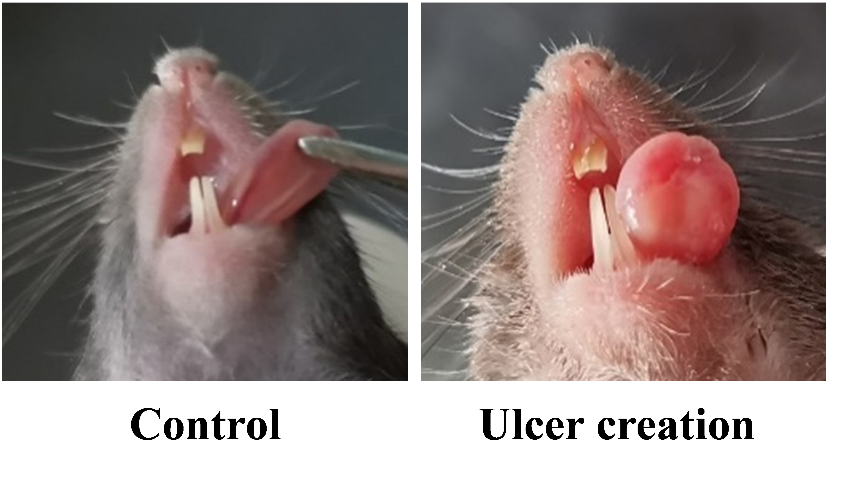


Supplementary Figure S6. Images of tongues before and after OM creation.


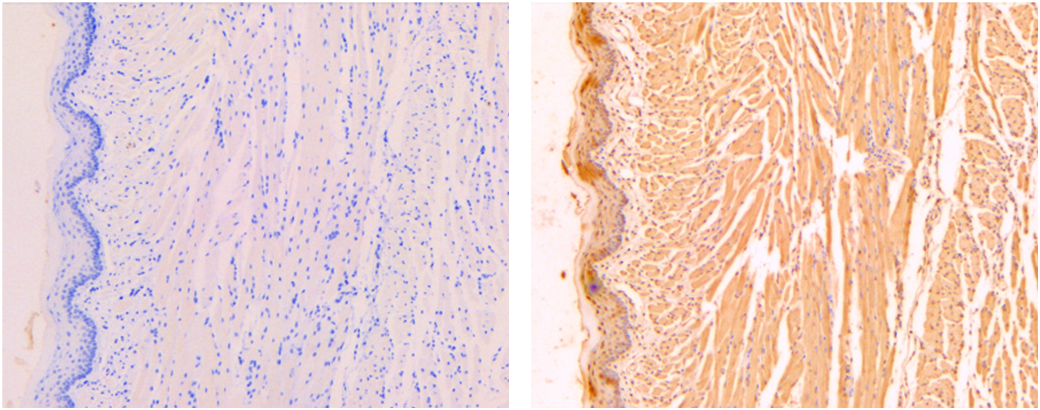


Supplementary Figure S7. Immunohistochemical staining of (left) Ly6G^+^ cells and (right) NF-κB p65 in normal tongues.
